# Commentary: GABA depolarizes immature neurons and inhibits network activity in the neonatal neocortex *in vivo*

**DOI:** 10.3389/fncel.2015.00478

**Published:** 2015-12-16

**Authors:** Yehezkel Ben-Ari

**Affiliations:** Institut National de la Santé et de la Recherche Médicale, Institut de Neurobiologie de la MéditerranéeNeurochlore, Marseille, France

**Keywords:** GABA depolarization, excitation-inhibition, immature neurons, cortical plate transient neurons, calcium current, anesthesia, *in vivo* imaging

Recording Cortical Plate (CP) neurons *in vivo* in pups shortly after birth, Kirmse et al. (2015) show that GABA exerts depolarizing but inhibitory actions. The technical achievements of this study are impressive but the following issues hamper the conclusions:

- Stress and *Anesthesia*: Isoflurane like nitrous oxide and other volatile anesthetics exert long lasting effects on calcium currents, GABAergic signals, and NMDA receptors (Krnjevic and Puil, 1997). Retinal waves are strongly affected by isoflurane (Ackman et al., 2012; Siegel et al., 2012). Stress molecule levels are highest at birth (Lagercrantz and Slotkin, 1986) and alter (Cl^−^)_I_(Ben-Ari, 2015) stressing the need to control the effects of anesthesia.
- *Exogenous GABA*: Cortical surface applications of GABA reflect extra synaptic not synaptic currents. The authors stress indeed that membrane potential, reversal potential of GABA, or timing and location of inputs are essential to determine whether excitatory or inhibitory effects dominate. Clearly, the validity of their conclusions is conditioned by determining the actions of GABA on PSCs (Gao and van den Pol, 2001; Tyzio et al., 2003).
- V rest and *GABA driving force*: Immature neurons have very high input resistance requiring single NMDA (or K^+^) channel recordings to determine V rest (Tyzio et al., 2003). Whether the cell-attached /current clamp method used by in this study are suitable for immature neurons requires a comparison with slice preparations.
- *CP neurons and developmental sequences*: The lack of neuronal identification is a major limitation as GABA excitatory actions are neuronal type and developmental stage dependent. Thus GABA excites interneurons but not adjacent pyramidal neurons in the same layer (Ben-Ari, 2014). Sub-plate and Cajal-Retzius neurons are depolarized and excited by GABA *in vitro* (Luhmann et al., 2014). Therefore, GABAergic depolarization and hyperpolarization might be restricted to some CP neurons.
- *In vitro damage and metabolic insult*: The ketone body and the traumatic explanation of the GABA sequence have been infirmed by many expert groups and not confirmed by a single one (Ben-Ari, 2014). Staley, Bernard and colleagues have recently used glucose perfused slices that they considered metabolically deprived and traumatized previously thereby infirming their own conclusions (Quilichini et al., 2012; Glykys et al., 2014). Conventional slices remain the best preparation we have to determine cellular mechanisms of the developing brain.
- *Bumetanide*: At the concentrations used by Kirmse et al. (50 μM), Bumetanide blocks NKCC1 and KCC2 limiting the conclusions on the polarity of GABA actions.
- *GDPs and ENOs*: Kirmse et al. suggest that their patterns are reminiscent of ENOs not GDPs. However, ENOS were blocked by bicuculline in their earlier study (Garaschuk et al., 1998); ENOs are also enhanced by pathological conditions challenging their physiological relevance (Allène et al., 2008).

Summing up, the GABA developmental sequence has been preserved throughout evolution in particular as far as the (Cl^−^)_*I*_ shift is concerned (Ben-Ari, 2014). The rise of calcium produced by depolarizing GABA is produced by a variety of signals including voltage gated calcium channels and NMDA receptors. Studies *in vivo* are of importance but at these early stages, stress and anesthetic agents cannot be readily dealt with. To some extent, the limitations of *in vivo* observations are far greater than those of neonatal slices. It is by combining *in vitro* and *in vivo* studies taking advantage of each that the maturation of brain patterns and their roles role in setting the operation of neuronal ensembles will be uncovered.

## Conflict of interest statement

The author declares that the research was conducted in the absence of any commercial or financial relationships that could be construed as a potential conflict of interest.
